# Protein Synthesis Inhibition Activity of Mesothelin Targeting Immunotoxin LMB-100 Decreases Concentrations of Oncogenic Signaling Molecules and Secreted Growth Factors

**DOI:** 10.3390/toxins10110447

**Published:** 2018-10-31

**Authors:** Salma El-Behaedi, Rebekah Landsman, Michael Rudloff, Emily Kolyvas, Rakan Albalawy, Xianyu Zhang, Tapan Bera, Keith Collins, Serguei Kozlov, Christine Alewine

**Affiliations:** 1 Laboratory of Molecular Biology, Center for Cancer Research, National Cancer Institute, National Institutes of Health, Bethesda, MD 20892-4264, USA; salma_el-behaedi@med.unc.edu (S.E.-B.); rlandsman@luc.edu (R.L.); michael.w.rudloff@vanderbilt.edu (M.R.); ekolyvas@mcw.edu (E.K.); Rakan.Albalawy@UToledo.edu (R.A.); xianyu.zhang@nih.gov (X.Z.); berat@mail.nih.gov (T.B.); 2 Center for Advanced Preclinical Research, National Cancer Institute at Frederick, National Institutes of Health, Frederick, MD 21702, USA; collinke@mail.nih.gov; 3 Center for Advanced Preclinical Research, Frederick National Laboratory for Cancer Research sponsored by the National Cancer Institute, P.O. Box B, Frederick, MD 21702, USA; kozlovse@mail.nih.gov

**Keywords:** immunotoxin, pancreatic cancer, VEGF, microenvironment, ubiquitination

## Abstract

LMB-100 is a mesothelin-targeted recombinant immunotoxin (iTox) that carries a modified *Pseuodomonas* exotoxin A (PE) payload. PE kills cells by inhibiting synthesis of new proteins. We found that treatment of pancreatic cancer cells with LMB-100 for 24–48 h did not change total protein level despite inducing protein synthesis inhibition (PSI). Further, increased levels of ubiquitinated proteins were detected, indicating that cells may have limited ability to compensate for PSI by reducing protein degradation. Together, these data suggest that PE depletes concentrations of a minority of cellular proteins. We used reverse phase protein array and Luminex assay to characterize this subset. LMB-100 decreased the abundance of 24 of 32 cancer-related proteins (including Bcl-x, Her2, Her3 and MUC16) without compensatory increases in other analytes. Further, cancer cells failed to maintain extracellular concentrations of cancer cell secreted growth factors (CCSGFs), including Vascular Endothelial Growth Factor (VEGF) following treatment with cytostatic LMB-100 doses both in culture and in mouse tumors. Decreased VEGF concentration did not change tumor vasculature density, however, LMB-100 caused tissue-specific changes in concentrations of secreted factors made by non-cancer cells. In summary, our data indicate that PSI caused by cytostatic LMB-100 doses preferentially depletes short-lived proteins such as oncogenic signaling molecules and CCSGFs.

## 1. Introduction

Pancreatic ductal adenocarcinoma (PDAC) has one of the poorest prognoses of any malignancy, and remains incurable in the majority of patients [1]. Combination chemotherapy regimens have resulted in improved outcomes over the last decade, but a dire need for new therapeutic options for this disease remains [2,3,4]. The dense desmoplastic stroma that surrounds cancer cells is considered one barrier to effective treatment of PDAC. This stromal reaction is initiated by hormones, growth factors and cytokines secreted by tumor cells (cancer cell secreted growth factors, CCSGFs) and results in a hypovascular, immunosuppressive microenvironment that impairs drug delivery and facilitates immune evasion. Multiple efforts are currently underway in the clinic to target components of the PDAC stroma or the heterotypic cellular signaling pathways that support these elements [5].

Recombinant immunotoxins (iTox) are antibody-based therapies which carry a bacterial toxin payload. LMB-100 (previously called RG7787) is a next generation iTox currently being tested in clinical trial (NCT02810418 and NCT03436732). LMB-100 contains a Fab fragment which targets the cancer cell surface antigen mesothelin (MSLN) fused in-line to a modified *Pseudomonas* exotoxin A (PE) payload [6,7,8]. The iTox is endocytosed following binding to MSLN, which is expressed in >90% of PDAC [9,10]. In the endocytic compartment, PE is cleaved from the Fab targeting region, and the toxin proceeds through the retrograde transport pathway to ultimately be secreted from the endoplasmic reticulum into the cytosol. PE is an enzyme which efficiently catalyzes an inhibitory, irreversible ADP-ribosylation of elongation factor-2 (EF-2). Since EF-2 is a non-redundant enzyme critical for protein translation, PE activity halts new protein synthesis in target cells [11]. This is a lethal insult in many cell types, including some PDACs [6,12].

The PE mechanism of action is unique amongst existing therapeutics for solid tumors: there are no approved therapies which target EF-2 or that specifically halt the production of new proteins by cancer cells. While the enzymatic activity of PE and the inhibitory effect of iTox on new protein synthesis have been well documented, it remains unclear how iTox treatment affects overall protein levels or those of individual proteins made by target cells. We examined the effect of LMB-100 treatment on cellular and secreted protein products of tumor cells in both cell culture and mouse models of PDAC. We found that PE-induced PSI depletes many proteins involved in oncogenic signaling both within the cell and outside in the tumor microenvironment.

## 2. Results

**Figure 1:**
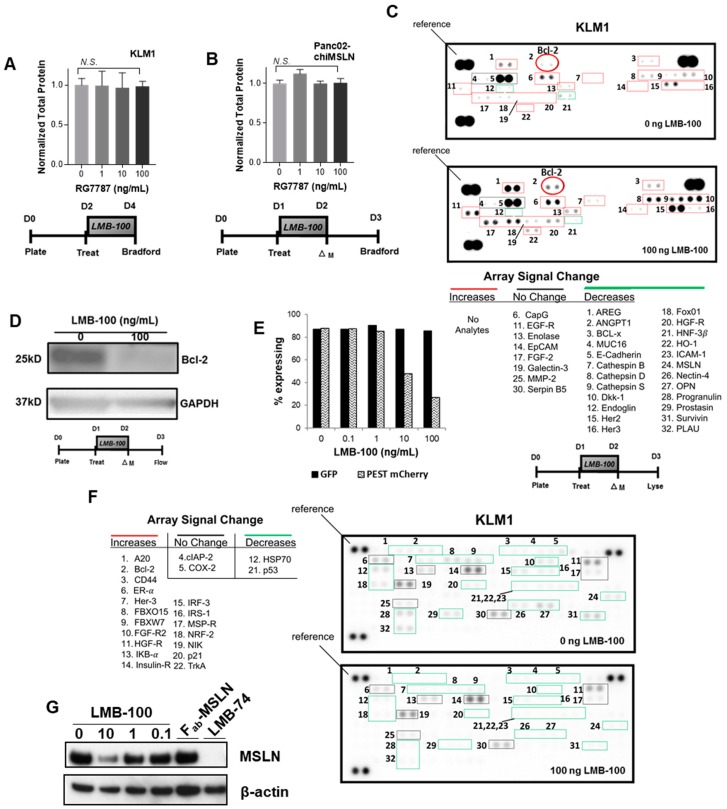
Effect of LMB-100 treatment on level of tumor cell proteins. (**A**) KLM1 cells were treated for 48 h with LMB-100 as per schema. Equal numbers of cells were lysed into a constant volume of lysis buffer and protein concentration assayed by standard colorimetric assay. (**B**) Panc02/chi-MSLN cells were treated with LMB-100 for 24 h as per schema. Protein concentration was assessed as described in (**A**). (**C**) KLM1 cells were treated as per schema and lysate assayed by RPPA for levels of the ubiquitinated forms of 49 protein analytes. “Reference” indicates control spots used to assess loading. (**D**) Immunoblot of lysates from (**C**) to assess level of Bcl-2. (**E**) KLM1 cells stably transfected with WT GFP and PEST-mCherry reporters driven off the same promoter through an IRES were treated with LMB-100 as per schema (Treat = start of LMB-100 treatment, ΔM = change medium). Percentage of cells with fluorescence above background in red and green channels was assessed by flow cytometry. (**F**) KLM1 cells treated as shown in schema were lysed and protein levels assayed by RPPA for levels of 84 cancer-related proteins. (**G**) Immunoblot of lysates from treated cells.

KLM1 is a human pancreatic cancer cell line produced by Kimura and colleagues by serial passaging of the PK1 line through mouse liver [13]. KLM1 cells express high levels of MSLN on the cell surface and are sensitive to MSLN-targeted iTox treatment [6]. Previously, it has been shown that LMB-100 causes a dose-dependent decrease in KLM1 new protein synthesis at 12 h with the maximal effect seen by 18 h post-initiation of treatment when using a 100 ng/mL dose. Even 24 h after washing away LMB-100, restoration of new protein synthesis is not observed [12]. We hypothesized that this prolonged protein synthesis inhibition (PSI) would lower total protein levels in cells. To test this, KLM1 cells were treated with LMB-100 for 48 h and then equal volumes of cell lysate produced from equal numbers of cells were assayed for protein concentration. Despite the PSI caused by LMB-100, cells maintained stable total protein levels even after 48 h of treatment with a high dose (100 ng/mL) of LMB-100 (Figure 1A). To determine whether this effect was specific for KLM1, we repeated this assay with a second pancreatic cancer cell line. Panc02 is a murine pancreatic cancer cell line developed by Corbett and colleagues [14]. Panc02 makes murine MSLN (mMSLN) but these cells are insensitive to LMB-100 since the iTox targets only human MSLN (hMSLN) and not mMSLN. To make a model cell line sensitive to LMB-100, Panc02 cells were stably transfected with an expression plasmid encoding a chimeric MSLN (chiMSLN) that is recognized by LMB-100 (Figure S1A). Surface expression of chiMSLN was verified by flow cytometry using an antibody targeted to the same epitope of hMSLN as LMB-100 (Figure S1B). As expected, expression of chiMSLN made Panc02 cells sensitive to LMB-100, resulting in a dose-dependent growth inhibition when cells were exposed to the iTox (Figure S1C). The treatment was cytostatic at best: total cell numbers measured 48 h after treatment were greater than baseline at all LMB-100 concentrations tested (Figure S1D). To confirm that LMB-100 treatment caused PSI in the Panc02-chiMSLN cells, we examined incorporation of low-dose puromycin into nascent peptide chains by SUnSET assay [15]. Puromycin incorporation was undetectable demonstrating successful PSI in this dose range (Figure S1E). Despite this, we found that Panc02-chiMSLN cells maintained stable total protein levels even after exposure to the highest dose of LMB-100 tested (Figure 1B). These data demonstrate that 48 h of exposure to LMB-100 inhibition does not reduce total protein levels in tumor cells despite profound inhibition of new protein synthesis. 

Both synthesis and degradation processes contribute to cellular proteostasis. We hypothesized that cells may slow down degradation processes to maintain stable total protein levels in the face of LMB-100-mediated PSI. Therefore, we compared levels of ubiquitinated proteins in lysate from KLM1 cells treated with vehicle or LMB-100 using a reverse phase protein array (RPPA) for ubiquitinated proteins. The RPPA allows assessment of relative levels of the ubiquitinated form of 49 proteins. Ubiquitination is a common signal used by cells to mark proteins for degradation. We anticipated that protein ubiquitination would decrease in treated cells, thereby slowing degradation of existing proteins to maintain proteostasis. Instead, we found increased levels of the ubiquitinated forms of 18 of the 22 array analytes detectable in our cells following LMB-100 treatment including fibroblast growth factor receptor-2 (FGF-R2) and insulin receptor (Figure 1C). Signal from two analytes decreased (p53 and heat shock protein (HSP70)), while cyclooxygenase-2 (COX2) and cellular inhibitor of apoptosis-2 (cIAP-2) signals remained unchanged. To confirm that increases in the ubiquitinated form correlated with decreased total protein level, we performed immunoblot for the anti-apoptotic protein Bcl-2 on the same lysates. We found that the Bcl-2 protein was decreased following LMB-100 treatment as expected (Figure 1D). These data are not consistent with our hypothesis that degradation is slowed following LMB-100 treatment and instead showed that cells preferentially increased ubiquitination to enhance degradation following LMB-100 exposure. 

Ubiquitination is one means by which cells can maintain “tight” post-translational control of critical proteins. It is expected that proteins with more rapid turnover, such as those subject to ubiquitination, would be disproportionately affected by LMB-100 treatment, while proteins with slower rates of degradation would maintain relatively stable levels. To test this, we needed to directly compare levels of a short-lived and a longer-lived protein that were synthesized by the cell in equal amounts. Therefore, we stably transfected KLM1 cells with a plasmid construct driving both green fluorescent protein (GFP) and PEST-mCherry fluorophore expression from the same promoter through use of an internal ribosome entry sequence (IRES). The half-lives of native GFP and mCherry exceed 24 h; however, addition of the PEST signal, a peptide sequence rich in proline (P), glutamic acid (E), serine (S) and threonine (T), targets proteins for rapid degradation, reducing protein half-life to less than 12 h [16]. We treated GFP + PEST-mCherry expressing cells with LMB-100 for 24 h then assessed the percent of cells with fluorescent signal by flow cytometry 24 h after this. We observed a dose-dependent decrease in mCherry expression with LMB-100 treatment, while GFP fluorescence did not change, consistent with our hypothesis that levels of short-lived proteins would be disproportionately affected by LMB-100 PSI (Figure 1E).

Recent quantitative proteomics studies have shown that proteins involved in signaling, cell adhesion, cell division, and regulation of cytokinesis have low expression levels and more rapid turnover [17,18]. Low abundance protein classes participate in many processes that are deranged in cancer cells. Therefore, we hypothesized that oncogenic signaling proteins would be relatively short-lived and therefore more susceptible to depletion by LMB-100 treatment. To examine whether LMB-100 could decrease individual levels of proteins involved in oncogenic processes, lysates from KLM1 cells treated with vehicle or LMB-100 were assayed by RPPA to examine levels of 84 cancer-related proteins. Of 32 analytes detectable in KLM1 lysate, 24 decreased following treatment, some to undetectable levels, and levels of eight remained unchanged. Two proteins that decreased in abundance, Her3 (Receptor Tyrosine Kinase ErbB3) and HGF-R (Hepatocyte Growth Factor Receptor/c-Met), had corresponding increased abundance of their ubiquitinated forms (Figure 1C). We observed no analytes with increased levels after LMB-100 treatment (Figure 1F). MSLN, the target of LMB-100, was one of the proteins found to decrease following iTox treatment. Immunoblot of treated lysates confirmed a dose-dependent decrease in MSLN expression following LMB-100 treatment, while treatment with the LMB-100 antibody fragment component lacking PE (Fab-MSLN) had no effect (Figure 1G). To further confirm the depletion is specific to PE rather than an effect of anti-MSLN antibody binding, we also treated cells with the LMB-74 iTox which has the same PE fragment as LMB-100 but an anti-transferrin receptor Fab for targeting. Similar to LMB-100, LMB-74 depleted total cell MSLN. In summary, LMB-100 treatment uses a PE-dependent process to decrease total protein levels of many cancer-related analytes without compensatory upregulation of others examined.

**Figure 2:**
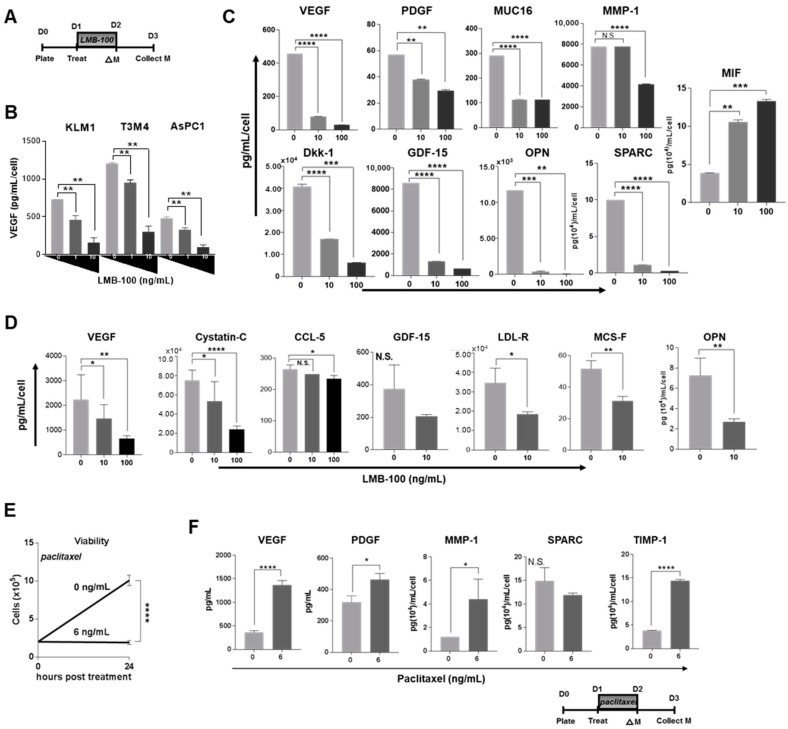
(**A**) LMB-100 treatment reduces levels of CCSGFs in conditioned medium, while paclitaxel increases it. For experiments in (**B**–**D**), the indicated pancreatic cancer cell lines were treated with LMB-100 for 24 h as per schema. Medium was replaced to stop treatment then collected 24 h later. (**B**) Conditioned medium from treated cells were assayed for VEGF using ELISA assay. (**C**) Conditioned medium from KLM1 cells was assayed for multiple CCSGFs by human analyte Luminex assay. (**D**) Conditioned medium from Panc02-chiMSLN cells was assayed for multiple CCSGFs by murine analyte Luminex assay. (**E**) Viable cells from triplicate wells were treated with paclitaxel for 24 h as shown in schema and then counted to assess viability. (**F**) Conditioned medium from KLM1 cells treated with paclitaxel were assayed for multiple CCSGFs by human analyte Luminex assay.

Tumor cells secrete growth factors and cytokines that promote their own proliferation and survival. The half-life of these soluble factors within the tumor microenvironment is presumed to be short due to uptake by target cells, diffusion into the circulation and surrounding tissues, and destruction by proteases. For instance, steady replenishment of pro-angiogenic VEGF-A by tumor cells is required to maintain local concentrations [19]. We hypothesized that the concentration of CCSGFs, such as VEGF, would be decreased following LMB-100 treatment due to tumor cell PSI caused by PE. Therefore, we treated pancreatic cancer cells with LMB-100 as per schema in Figure 2A, then examined levels of VEGF in conditioned medium by ELISA. We found that LMB-100 treatment caused a dose-dependent decrease in conditioned medium VEGF concentration in KLM1, T3M4 and AsPC1 pancreatic cancer cell lines (Figure 2B). To determine whether LMB-100 could suppress levels of other CCSGFs, we repeated this assay using a human analyte Luminex plate which allowed us to simultaneously assess for changes in the protein concentration of multiple analytes. In eight of nine detectable analytes, we observed a dose-dependent concentration decrease in conditioned medium following LMB-100 treatment. These included VEGF, platelet-derived growth factor (PDGF), mucin-16 (MUC16), matrix metalloproteinase-1 (MMP-1), Dickkopf-related protein 1 (Dkk-1), growth/differentiation factor 15 (GDF-15), osteopontin (OPN), and osteonectin/secreted protein acidic and rich in cysteine (SPARC) (Figure 2C). Conversely, LMB-100 treatment increased conditioned medium levels of one analyte, macrophage migration inhibitory factor (MIF), consistent with prior reports that pre-synthesized pools of this protein are released by some cells under stress [20]. This experiment was repeated using Panc02-chiMSLN cells. Once again, a dose-dependent decrease in concentration of six of seven analytes (murine isoforms of VEGF, Cystatin-C, Chemokine C-C motif ligand 5 (CCL-5), low density lipoprotein receptor (LDL-R), macrophage colony stimulating factor (MCS-F), and OPN) assayed by mouse analyte Luminex was observed in conditioned medium from cells treated with LMB-100, and no statistically significant difference in concentration was detected in the seventh (murine GDF-15) (Figure 2D). By comparison, treatment of KLM1 cells with the chemotherapy agent paclitaxel given at a cytostatic dose (Figure 2E), resulted in increased conditioned medium concentration of four of five analytes examined (VEGF, PDGF, MMP-1, and Tissue inhibitor of metalloproteinases-1 (TIMP-1)), and did not change the concentration of the fifth (SPARC) (Figure 2F). These data demonstrate that LMB-100 treatment reduces concentrations of CCSGFs in conditioned medium of cultured tumors cells, unlike treatment with paclitaxel chemotherapy.

**Figure 3:**
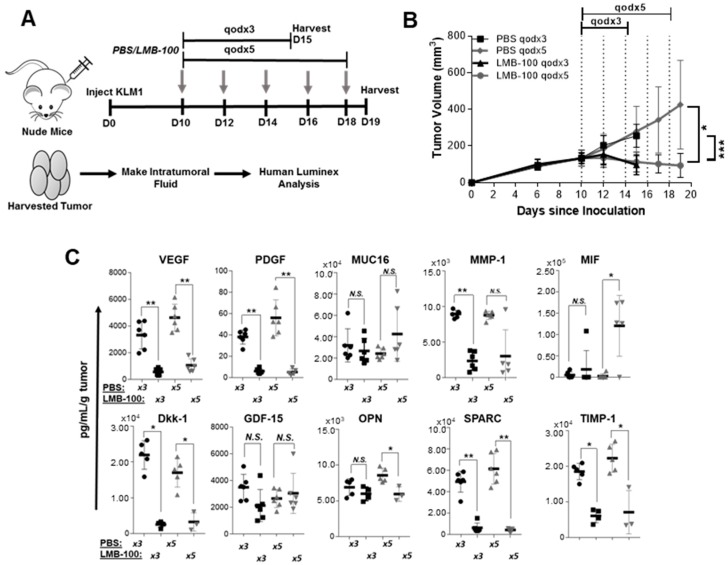
LMB-100 reduces levels of CCSGFs in ITF of nude mouse xenograft model. (**A**) Treatment schema. Nude mice bearing subcutaneous KLM1 tumors of ~100 mm^3^ were randomized to treatment with PBS vehicle or LMB-100 (2.5 mg/kg) given IV every other day for three (qodx3) or five (qodx5) doses. Mice were euthanized 24 h after final treatment and tumors harvested for extraction of ITF. (**B**) Tumor growth curve of LMB-100 and PBS treated animals. LMB-100 treatment resulted in a statistically significant decrease in tumor burden compared to PBS in both qodx3 and qodx5 treatment groups. (**C**) ITF from harvested tumors was assayed for multiple CCSGFs by human analyte Luminex assay.

LMB-100 PSI should also decrease concentrations of CCSGFs in intratumoral fluid (ITF) following in vivo LMB-100 treatment. Therefore, aythmic nude mice bearing KLM1 subcutaneous tumors were treated with three or five doses of LMB-100, and then 24 h later the mice were euthanized and ITF collected from harvested tumors (Figure 3A). We utilized a dose and schedule of LMB-100 that halted tumor growth but did not significantly decrease tumor size from pre-treatment baseline (Figure 3B). Using the human-specific Luminex assay from our cell culture experiments, we again detected concentration decreases in seven of 10 detectable analytes, including VEGF. MIF concentration increased in ITF, as seen in conditioned medium from treated cells. No change was detected in GDF-15 concentration. Similar results were seen using the LMB-74 anti-transferrin iTox (data not shown). These data demonstrate that LMB-100 treatment reduces levels of many CCSGFs in vivo.

**Figure 4:**
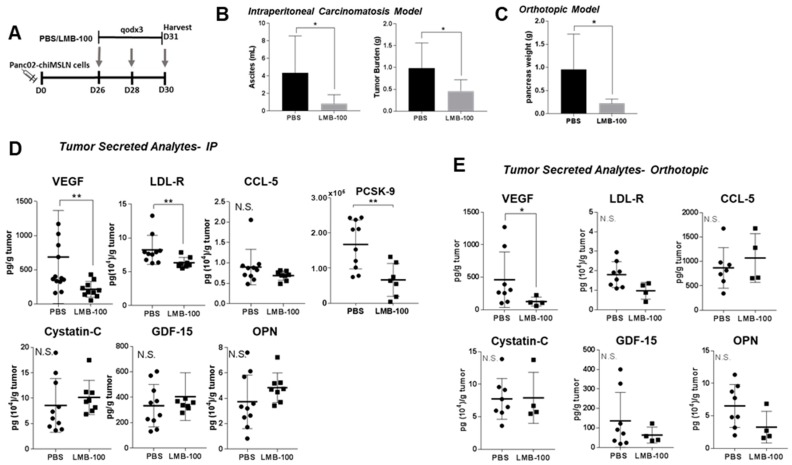
LMB-100 reduces levels of CCSGFs in ITF of syngeneic orthotopic and IP metastasis models. (**A**) Treatment schema. C57Bl/6-CAG > hMSLN mice were inoculated IP with Panc02-chiMSLN tumor cells. Tumors were grown for ~3.5 weeks before initiation of treatment with LMB-100 (2.5 mg/kg) given IV every other day for three (qodx3) doses or to equivolume PBS vehicle given on the same schedule. Mice were euthanized 24 h after final treatment and tumors harvested for extraction of ITF. (**B**) Ascites volume and total IP tumor burden in treated animals were measured. (**C**) C57Bl/6-CAG>hMSLN mice were inoculated into the pancreas with Panc02-chiMSLN tumor cells, then tumors grown for six weeks. Treatments were performed as described in (**A**). Pancreas weight was measured at necropsy of treated animals to assess tumor burden. (**D**,**E**) ITF from harvested tumors was assayed for multiple CCSGFs by mouse analyte Luminex assay. PCSK-9 level was assessed by ELISA since this analyte was not available on Luminex platform.

LMB-100 specifically induces PSI in MSLN-expressing tumor cells but has no activity against the cells in the tumor microenvironment because they do not express hMSLN. Consequently, loss of CCSGF production by cancer cells due to LMB-100 PSI could be compensated for by increased production of CCSGFs by other hMSLN-negative cell types within tumors such as activated fibroblasts, immune cells or endothelial cells. In the KLM1 xenograft model, the Human Luminex assay can distinguish cancer cell from host contribution to the ITF milieu since detection of many analytes is human-specific. To determine whether the effect of LMB-100 treatment is strong enough to change the composite (cancer cell + other host contribution) concentrations of CCSGFs, we repeated our in vivo assay in a syngeneic model, where composite concentrations of the analytes could be examined since all contributing cells produce murine isoform. Panc02-chiMSLN cells were inoculated into the abdominal cavity of syngeneic wild-type (WT) C57Bl/6 mice. However, in our pilot experiments, we observed limited tumor growth, presumably due to host intolerance of the chiMSLN transgene. Therefore, we utilized a transgenic C57Bl/6 mouse line (C57Bl/6-CAG) engineered to express hMSLN in addition to native mMSLN. Using a moderate dose of LMB-100 (40 µg flat dose), we found that three LMB-100 treatments (see schema in Figure 4A) resulted in a statistically significant decrease in both ascites volume and total tumor burden in an intraperitoneal (IP) tumor model (Figure 4B) and decreased pancreas weight in an orthotopic model (Figure 4C). In the IP model, a statistically significant decrease in murine VEGF, LDL-R and PCSK-9 concentrations in ITF was observed, however, no change in ITF concentration was identified for three other analytes (murine CCL-5, cytostatin-C, and OPN), which had decreased in previous cell culture experiments (Figure 4D). Similarly, in the orthotopic model, we observed a statistically significant decrease in VEGF concentration in ITF fluid, and a decrease in LDL-R and OPN concentrations which did not reach statistical significance (Figure 4E). No change in ITF concentration was observed in two other analytes with decreased concentrations following LMB-100 treatment in cell culture experiments (CCL-5, cystatin-C). These data demonstrate that total local tumor concentrations of selected CCSGFs, such as VEGF, can be significantly reduced by LMB-100.

**Figure 5:**
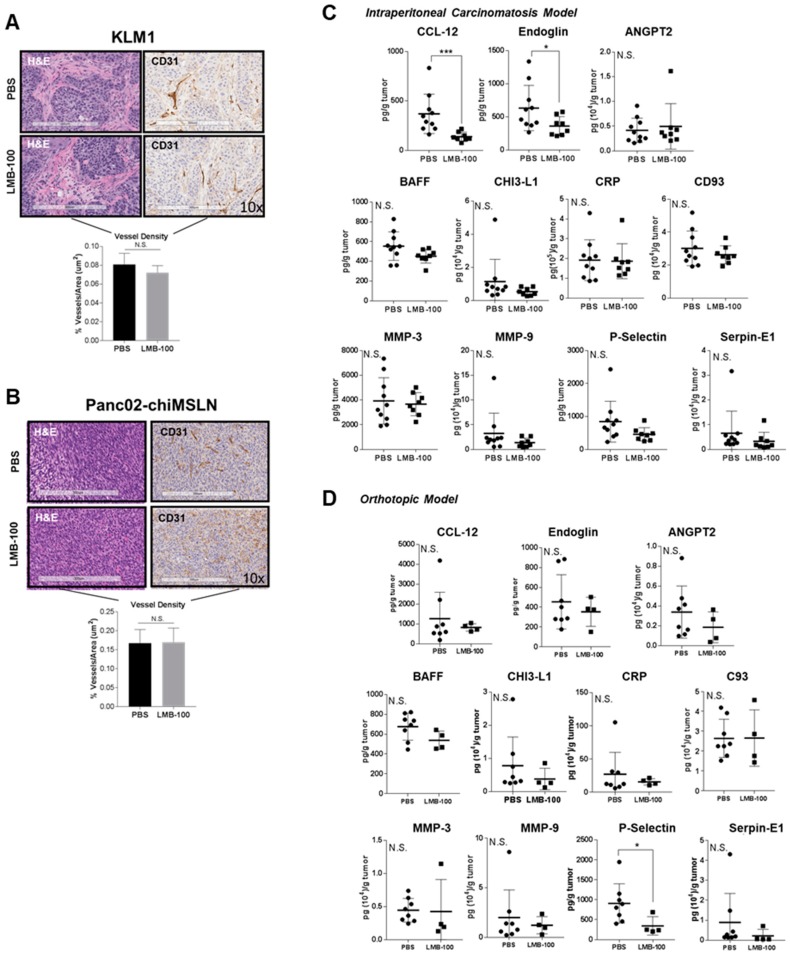
Changes in CCSGFs are insufficient to cause detectable changes in tumor vascular density or in secreted protein milieu. Formalin-fixed paraffin embedded KLM1 tumor samples from mice treated as described in Figure 3 (**A**), and Panc02-chiMSLN IP tumor samples treated as described in Figure 4 (**B**) were both stained for CD31, a marker of vascular density. The percent vessel area in four mice per group was assessed by a pathologist blinded to treatment status at Pathology/Histotechnology Laboratory Core Facility (NCI, Frederick, MD). (**C**,**D**) ITF from harvested tumors was assayed for multiple CCSGFs by mouse analyte Luminex assay.

Secretion of growth factors and cytokines by pancreatic cancer cells both induce and maintain the unique pancreatic cancer microenvironment. LMB-100 treatment suppresses local tumor concentration of CCSGFs such as VEGF, and therefore LMB-100 treatment might remodel the tumor microenvironment. Since VEGF influences angiogenesis, we examined vascular density in treated tumors. No difference in vascular density was observed in the KLM1 subcutaneous tumors (Figure 5A) or in Panc02-chiMSLN IP tumors following LMB-100 treatment (Figure 5B). Other parameters including mean vessel area and diameter were also unchanged (data not shown). LMB-100 treatment was insufficient to remodel tumor vessels over the short time-course of our experiment, despite causing a reduction in VEGF levels.

We next considered whether the changes in CCSGF concentration induced by LMB-100 might alter chemical crosstalk between cells in the microenvironment and lead to changes in the local concentration of ITF proteins not secreted by tumor cells. Therefore, we assayed ITF from Panc02-chiMSLN tumors for levels of 11 analytes undetectable in conditioned medium from these cells, yet detectable in ITF extracted from tumors. There was a statistically significant decrease in concentration of CCL-12 (chemokine C-C motif ligand 12) and endoglin in IP tumor ITF that was not observed in orthotopic tumor ITF samples, and a trend to decreased concentration of P-selectin in IP tumor ITF, that did reach statistical significance in the orthotopic model (Figure 5C,D). No significant changes in concentration were observed for any of the eight other analytes measured. In conclusion, decreases in CCSGFs caused by LMB-100 altered concentrations of selected factors secreted by non-tumor cells in a tissue-dependent fashion, but were insufficient to alter ITF concentrations of most analytes.

## 3. Discussion

We have shown that LMB-100 treatment depletes levels of many tumor-secreted proteins and short-lived proteins including those involved in oncogenic signaling. In fact, LMB-100 treatment modified the cancer cell secretome to reduce concentrations of selected cytokines and growth factors within tumor fluid even when using cytostatic doses of the iTox. By contrast, treatment with the chemotherapy drug paclitaxel at cytostatic doses resulted in increased concentrations of several CCSGFs that support tumor cell proliferation. This result reflects the unique mechanism of iTox action and may explain why LMB-100 can synergize with taxanes and other anti-cancer therapeutics [12,21,22]. 

We found that doses of LMB-100 that do not kill cells but do cause PSI modulate the cancer cell secretome, resulting in decreased extracellular concentrations of most CCSGFs that we studied (see Table 1). In all models tested, reduced VEGF concentration was observed. Moreover, impaired tumor cell secretion of VEGF was sufficient to lower total levels of VEGF within tumor fluid, suggesting that tumor cells are the primary contributors of VEGF to the tumor microenvironment. Over the timescale that we studied, we observed no changes in tumor vascular density, a hallmark of VEGF activity, despite the observed reduction in tumor fluid VEGF concentration following iTox treatment. Regulation of vascular dynamics by VEGF is complicated and relies not only on local concentration, but also on concentration gradient to stimulate migration of endothelial cells [23], which we did not measure in our experiments. Further, secreted VEGF can be immobilized in tumor extracellular matrix components to maintain this gradient [24]. Future studies would be required to examine these dynamics in greater detail.

We expected that cells might try to compensate for the halt in new protein synthesis caused by iTox by slowing down degradation processes. Instead, our data indicate that protein ubiquitination was almost universally increased following LMB-100 treatment, a novel finding. While it is beyond the scope of this study to confirm that increased ubiquitination of these targets equated with their increased degradation, we did see that three of the 18 array analytes with amplified ubiquitination (Her3, HGF-R and Bcl-2) had decreases in protein abundance following LMB-100 treatment. Future studies would be required to determine whether slowing ubiquitin-mediated protein degradation using a proteasome inhibitor could prevent iTox-induced decreases in protein abundance, and if so, may also provide insight into whether depletion of one or more ubiquitinated proteins is the direct mechanism causing iTox-mediated cell death.

Our data show that levels of many cancer-related proteins were diminished following LMB-100 treatment without evidence for compensatory upregulation in others. Concentration decreases were observed for two-thirds of detectable analytes on a standard Oncology RPPA following LMB-100 treatment, and no analytes increased. GFP + PEST-mCherry reporter assay demonstrated that short-lived proteins were most affected by LMB-100-mediated protein synthesis inhibition, similar to what has been seen previously with the chemical protein synthesis inhibitor cychloheximide. Our RPPA data corroborate this observation. Schwanhäusser and colleagues analyzed the half-lives of over 5000 proteins made by NIH3T3 fibroblast cells [17]. The half-lives of five proteins with decreases in abundance following LMB-100 treatment (Bcl-x, Fox01, HO-1, survivin, and PLAU) were reported and ranged from 4.9 (PLAU) to 20.8 h (Bcl-x), which is substantially below the 48-h median for the complete dataset. Half-lives of four proteins with no change in abundance following LMB-100 treatment were also reported: CapG, enolase, galectin-3, and EGF-R had measured half-lives of 266, 214, 61, and 12.7 h, respectively. Notably, three of the four exceeded the 48-h median (EGF-R which is known to have ligand-dependent stability was the exception) [25]. These data support our finding that short half-life proteins are most sensitive to depletion following iTox treatment.

At the outset, we expected that LMB-100 treatment would decrease total cellular protein levels due to PE-mediated protein synthesis inhibition. Others have previously shown that the most stable cellular proteins, those involved in housekeeping processes such as translation, protein metabolism, glycolysis, purine metabolism, and the citric acid cycle, are the most abundant within the cell, constituting ~50% of the cellular proteome [17,18]. By contrast, short half-life proteins such as those important in signaling and cell adhesion account for ~10% of the quantitative proteome. This means that even a 50% decrease of all short-lived proteins would be expected to change total bulk protein levels by only ~5%. Given these metrics, it is unsurprising that LMB-100 treatment did not decrease total cellular protein levels over the time course that we studied.

In summary, we have shown for the first time that PE-based iTox decreases the cellular and whole tumor concentrations of a broad range of proteins involved in oncogenic signaling without detectable compensatory upregulation of others. In addition, immunotoxin treatment depletes local tumor levels of many growth factors, cytokines and proteases produced by tumor cells, and does so even when administered at low doses insufficient to kill target cells. 

## 4. Materials and Methods 

### 4.1. Cell Culture and Reagents 

Human pancreatic cancer KLM1 and T3M4 cells were provided by Ira Pastan and Mitchell Ho (both of NCI, Bethesda, MD, USA), respectively. AsPC1 was purchased from ATCC. Panc02 cells were acquired from the NCI Frederick repository. Identity of all cell lines was confirmed by STR testing. Cells were cultured in RPMI 1640 medium (Gibco, Thermo Scientific, Waltham, MA, USA) supplemented with L-Glutamine (2 mmol/L), penicillin (100 U), streptomycin (100 µg), and 10% FBS (Hyclone, Thermo Scientific). All cells were maintained in a 5% CO_2_ at 37 °C and tested free of mycoplasma. The MSLN targeting iTox, LMB-100, was manufactured by F. Hoffman La Roche and provided for these studies through a Collaborative Research and Development Agreement. LMB-74 was made as described previously [12].

### 4.2. Generation of Panc02-chiMSLN Cell Line 

A synthetic 213bp DNA fragment containing exon 11 sequence of hMSLN cDNA was cloned between BstEII and SacII restriction sites in the mouse ORF to replace the corresponding murine exon 10 sequence. This substitution resulted in production of a chiMSLN containing the mature mMSLN polypeptide with 64 amino acid residues substituted by a corresponding homologous segment of the human protein (Figure S1A). The chimeric cDNA fragment was cloned into a neomycin-resistant expression vector under the transcription control of RNA Polymerase II promoter. Panc02 cells were transfected with PolII-chiMSLN construct using Nucleofection (Lonza, Walkersville, MD, USA) with DNA Nucleofector Kit R as per manufacturer’s recommendations. Following 24-h recovery, cells were selected in 0.5 mg/mL of G418 for 72 h. Surviving cells were analyzed for surface expression of the chiMSLN and subsequently twice sorted by flow cytometry to obtain a clonal population. Panc02-chiMSLN cells were cultured with 50 µg/mL gentamicin to maintain chimeric mouse-human MSLN (chiMSLN) expression.

### 4.3. Cytotoxicity Assay 

Cells were plated in 96-well plates at 5000 cells/well on Day 0 and treated with iTox 24 h later. Relative cell viability was measured using the Cell Counting Kit-8 WST assay (Dojindo Molecular Technologies, Inc., Rockville, MD, USA). Values were normalized between 0% viability for treatment with positive control drug (which produced complete cell killing), and 100% viability for addition of medium alone.

### 4.4. Cell Lysate, Protein Quantification and Immunoblotting 

Cells were harvested by scraping, washed in DPBS lacking calcium and magnesium (Gibco, 14190-144), and lysed in RIPA buffer (20 mM Tris, pH 7.5, 150 mM NaCl, 1 mM EDTA, 1% Triton X-100, and 1% Tween-20) containing protease/phosphatase inhibitors (Thermo Scientific). Total protein concentration was determined using Pierce BCA Protein Assay Kit (Thermo Scientific). Proteins were separated by SDS-PAGE, transferred to PVDF membrane, and blocked in 5% milk solution. The following antibodies were used: goat anti-human/mouse anti-Bcl-2 (AF810, R&D Systems, Minneapolis, MN, USA) and mouse anti-human anti-puromycin (MABE343, Millipore Sigma, St. Louis, MO, USA).

### 4.5. Collection of Cell Culture Conditioned Medium

KLM-1, T3M4, and AsPC1 cells were plated at equal densities and allowed to attach overnight, then exposed to LMB-100. Panc02-chiMLSN cells were plated at dose-specific density (2.5 × 10^4^ cells/well for 0 or 10 ng/mL LMB-100 treatment, 1.0 × 10^5^ for 10 ng/mL, or 2.5 × 10^6^ 100 ng/mL) to allow for normalization and cultured overnight before treatment with LMB-100 for 24 h. Conditioned medium was collected 24 h after washout of LMB-100, aliquoted and frozen at −80 °C until ready to be analyzed by ELISA or Luminex.

### 4.6. Flow Cytometry 

Cells were harvested using trypsin then resuspended in PBS. For experiments with cells co-expressing GFP and PEST-mCherry, compensation was performed using cells transfected with GFP or mCherry alone. For assessment of MSLN expression, cells were incubated with mouse anti-human MSLN MN-1 or MORAb009 humanized anti-human MSLN antibody for 30 min on ice followed by PE conjugated goat anti-mouse IgG F(ab’)_2_ fragment (Jackson Immunoresearch, West Grove, PA, USA) staining for 30 min. Cells were re-suspended in FACS buffer (5% FBS in PBS) prior to analysis on a BD FACS Calibur. 

### 4.7. Establishment of C57BL/6 Transgenic Mice Expressing Human Mesothelin 

Transgenic C57Bl/6-CAG>hMSLN mice were made as described previously [26]. Briefly, a cDNA that consists of full-length *MSLN* under the control of a CAG promoter was produced. The pronucleus of fertilized oocytes from C57Bl/6 mice were microinjected with the plasmid. Founder animals carrying a h*MSLN* transgene were identified by Southern blot analysis followed by PCR screening to establish founder lines. 

### 4.8. Mouse Tumor Experiments 

All animal experiments were performed in accordance with institutional guidelines and approved by the institution Animal Care and Use Committee. For tumor experiments utilizing KLM1, female 6–8-week-old athymic nude mice (Charles River, (location withheld to conceal author identity)) were inoculated subcutaneously with 3 × 10^6^ cells in 4.0 mg/mL Matrigel (Corning) in RPMI 1640 (Gibco) with no additives. Tumor size was measured in two dimensions by digital calipers and tumor volume was calculated using the formula: 0.4 × width^2^ × length. Once KLM1 xenografts tumors had reached a size of ~100 mm^3^ (10 days), mice were randomized into treatment groups. Mice were euthanized 24 h after the final treatment and tumor harvested. 

For tumor experiments using Panc02-chiMSLN cells, 6–10-week-old C57Bl/6-CAG mice were injected in the IP cavity with 1 × 10^6^ cells in RPMI 1640 (Gibco) with no additives. Mice were monitored for 3–4 weeks until the IP tumor became palpable, then randomized into treatment groups. For orthotopic experiments, a small incision was made in anesthetized mice to expose the pancreas and 1 × 10^5^ cells were injected into the pancreatic tail. Treatment was initiated ~6 weeks later. All mice were euthanized 24 h after the last treatment, ascites was withdrawn and measured, and all visible tumor in the IP cavity was harvested by dissection and weighed. 

### 4.9. ITF Preparation

Harvested tumors were suspended in 750 µL of RPMI 1640 (Gibco) with no additives. Tumor was cut into fine pieces using surgical scissors, re-suspended in an additional 750 µL of RPMI, and tumor debris spun down at 1000× *g* for 3 min at 4 °C. All supernatant was recovered and frozen at −80 °C in aliquots until analysis.

### 4.10. ELISA, Luminex and RPPA Assays 

Human VEGF ELISA kit was purchased from ThermoFisher. MMP-9, murine OPN, murine PCSK9 ELISA kits, Proteome Profiler Human Ubiquitin and XL Oncology reverse phase protein arrays were purchased from R&D Systems. Changes in protein expression on RPPA were evaluated by eye as per manufacturer’s instructions. Custom magnetic bead Luminex assays were purchased from R&D Systems. To choose Luminex analytes to assay, pilot vehicle and LMB-100 treated murine samples were tested on Proteome Profiler Mouse XL Cytokine Array (R&D Systems), to identify detectable targets. Luminex analytes were then chosen based on availability on the Luminex platform and compatibility with other potential analytes. Available analytes for the mouse and human arrays differed, such that the CCSGFs that we profiled were not identical for the two species, however, analytes that were available for both species and expressed by our models were assayed wherever possible. A MagPix device using Xponent software was used to analyze Luminex assays (R&D Systems). 

### 4.11. Histological Analyses 

Tumor samples were sent to (institutional veterinary histology core) Laboratory core facility for all histologic studies and analysis. Core pathologists used Aperio Microvessel Analysis Algorithm for assessment of vasculature. 

### 4.12. Statistics

GraphPad Prism 7 software and Microsoft Excel were used for all statistical analysis and graphing. Data are presented as averages with error bars representing standard deviations unless stated otherwise. ANOVA was used for multiple comparisons followed by post-hoc *t*-test. Two-tailed Student’s *t*-test was used for two group comparison of conditioned medium samples and histological analysis. Mann–Whitney test was used for two group comparisons of in vivo ITF quantification. All experiments were confirmed by repeat. For all figures: * *p* ≤ 0.05, ** *p* ≤ 0.01, *** *p* ≤ 0.001, **** *p* ≤ 0.0001.

## Figures and Tables

**Table 1:** Summary of Treatment Effect on Analyte Concentration.

Human Panel: CCSFs	Paclitaxel	LMB-100	
	Cell Culture	Cell Culture	Subq Model
Dkk-1	NA	−	−
GDF-15	NA	−	0
MIF	NA	+	+
MMP-1	+	−	−
MUC16	NA	−	0
OPN	NA	−	−
PDGF	+	−	−
SPARC	0	−	−
TIMP-1	+	NA	−
VEGF	+	−	−
Mouse Panel: CCSFs	Cell Culture	IP Model	Orthotopic Model
CCL-5	−	0	0
Cystatin-C	−	0	0
GDF-15	0, trend -	0	0, trend −
LDL-R	−	−	0, trend −
MCS-F	−	NA	NA
OPN	−	0, trend +	0, trend −
PCSK-9	NA	−	NA
VEGF	−	−	−
Mouse Panel: microenvironment factors	Cell Culture	IP Model	Orthotopic Model
CCL-12	ND	−	0
ANGPT2	ND	0	0
BAFF	ND	0	0
CD93	ND	0	0
CHI3-L1	ND	0	0
CRP	ND	0	0
endoglin	ND	−	0
MMP-3	ND	0	0
MMP9	ND	0	0
P-selectin	ND	0	−
Serpin-E1	ND	0	0

+, increase; −, decrease; 0, no change; NA, not assayed; ND, not detected.

## Supplementary Materials

The following are available online at http://www.mdpi.com/2072-6651/10/11/447/s1. Figure S1: Panc02-chiMSLN cell model.
