# Supplementary material for: Protein Synthesis Inhibition Activity of Mesothelin Targeting Immunotoxin LMB-100 Decreases Concentrations of Oncogenic Signaling Molecules and Secreted Growth Factors
Source: Toxins (Basel). 2018 Oct 31;10(11):447. doi: 10.3390/toxins10110447 (PMC6267581; doi:10.3390/toxins10110447)
Supplement: Supplementary file 1 [file toxins-10-00447-s001.pdf]

Supplementary Materials: Protein Synthesis Inhibition Activity of Mesothelin Targeting Immunotoxin LMB-100 Decreases Concentrations of Oncogenic Signaling Molecules and Secreted Growth Factors

Salma El-Behaedi, Rebekah Landsman, Michael Rudloff, Emily Kolyvas, Rakan Albalawy, Xianyu Zhang, Tapan Bera, Keith Collins, Serguei Kozlov and Christine Alewine

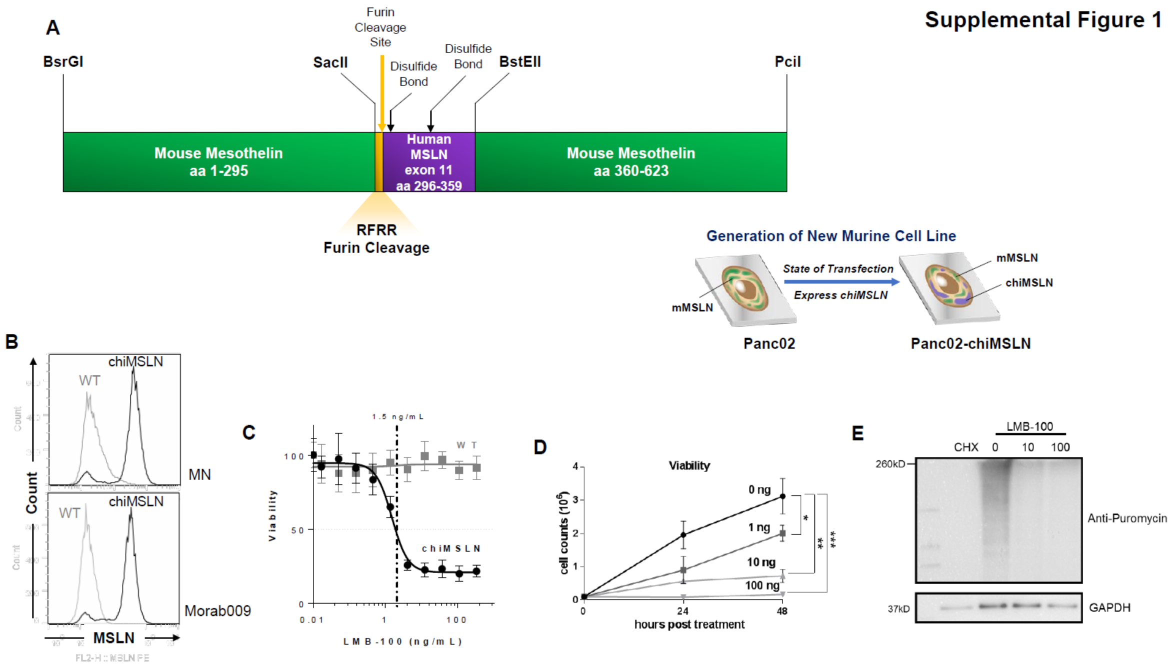

Figure S1. Panc02-chiMSLN cell model. (**A**) Schema of chiMSLN construct. (**B**) WT Panc02 and Panc02-chiMSLN cells were treated with a concentration curve of LMB-100 and viability was assessed by colorimetric assay after 72 hours. (**C**) WT Panc02 and Panc02-chiMSLN cells were incubated with anti-hMSLN monoclonal antibodies that recognize chiMSLN (Morab009 or MN) followed by appropriate phycoerythrin-labeled secondary, then surface expression of chiMSLN was assessed by flow cytometry. (**D**) Viable Panc02-chiMSLN cells were counted 24 and 48 hours after initiation of LMB-100 treatment. (**E**) Panc02-chiMSLN cells were treated with LMB-100 or cycloheximide (positive control for protein synthesis inhibition) for 24hrs, pulse labeled with low dose puromycin, then lysed and immunoblotted with anti-puromycin antibody to assess for protein synthesis inhibition. Shown is representative blot of triplicate samples.
